# An Account of Diseases in an Eastern District of London from August 20, to September 20, 1809

**Published:** 1809-10

**Authors:** 


### *An Account of Diseases in an Eastern District of London from August 20, to September 20, 1809.*

| | |
|---|---|
| **ACUTE DISEASES.** | |
| Typhus | 2 |
| Febris Intermittens | 1 |
| Erysipelas | 1 |
| Rheumatismus Acutus | 2 |
| **CHRONIC DISEASES.** | |
| Tussis | 8 |
| Dyspnœa | 4 |
| Tussis cum Dyspnœa | 10 |
| Hæmoptysis | 2 |
| Hydrothorax | 3 |
| Hæmatemesis | 2 |
| Syncope | 2 |
| Hysteria | 1 |
| Hypochondriasis | 3 |
| Fluor Albus | 5 |
| Dysuria | 3 |
| Dysmenorrhœa | 5 |
| Rheumatismus Chronicus | 5 |
| **PUERPERAL DISEASES.** | |
| Mastodynia | 3 |
| Lactea | 2 |
| Menorrhagia Lochialis | 4 |
| Dysuria | 3 |
| **INFANTILE DISEASES.** | |
| Erysipelas Infantile | 2 |
| Tabes Mesenterica | 2 |
| Convulsio | 1 |
| Vermes | 4 |

